# Application of selected reaction monitoring mass spectrometry to field-grown crop plants to allow dissection of the molecular mechanisms of abiotic stress tolerance

**DOI:** 10.3389/fpls.2013.00020

**Published:** 2013-02-13

**Authors:** Richard P. Jacoby, A. Harvey Millar, Nicolas L. Taylor

**Affiliations:** Australian Research Council Centre of Excellence in Plant Energy Biology and Centre for Comparative Analysis of Biomolecular Networks, The University of Western AustraliaCrawley, WA, Australia

**Keywords:** abiotic stress, molecular breeding, wheat, barley, rice, selected reaction monitoring, proteomics

## Abstract

One major constraint upon the application of molecular crop breeding approaches is the small number of genes linked to agronomically desirable traits through defined biochemical mechanisms. Proteomic investigations of crop plants under abiotic stress treatments have identified many proteins that differ in control versus stress comparisons, however, this broad profiling of cell physiology is poorly suited to ranking the effects and identifying the specific proteins that are causative in agronomically relevant traits. Here we will reason that insights into a protein’s function, its biochemical process and links to stress tolerance are more likely to arise through approaches that evaluate these differential abundances of proteins and include varietal comparisons, precise discrimination of protein isoforms, enrichment of functionally related proteins, and integration of proteomic datasets with physiological measurements of both lab and field-grown plants. We will briefly explain how applying the emerging proteomic technology of multiplexed selective reaction monitoring mass spectrometry with its accuracy and throughput can facilitate and enhance these approaches and provide a clear means to rank the growing cohort of stress responsive proteins. We will also highlight the benefit of integrating proteomic analyses with cultivar-specific genetic databases and physiological assessments of cultivar performance in relevant field environments for revealing deeper insights into molecular crop improvement.

## CROP LOSSES DUE TO ABIOTIC STRESS AND THE PROMISE OF APPLYING MOLECULAR TECHNIQUES TO CROP IMPROVEMENT

**FIGURE 1:**
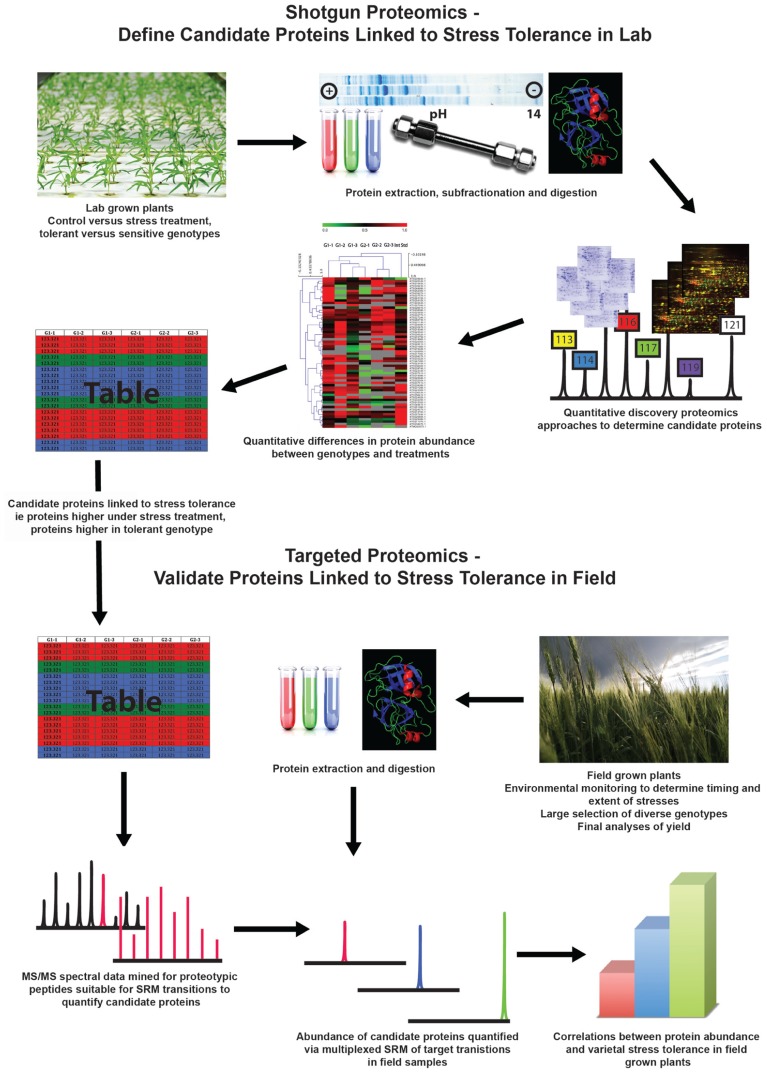
**Proposed workflow that combines laboratory-based discovery proteomics followed by selected reaction monitoring mass spectrometry of field-grown plants to gain more relevant information to advance both gene discovery and gene evaluation for crop improvement programs**.

Analyses by agricultural researchers and humanitarian organizations have continually found that abiotic stresses such as drought, extreme temperatures, and unfavorable soil conditions are responsible for significant decreases in crop yield and can have adverse economic and nutritional consequences for local populations (Boyer, 1982; Mittler, 2006; Witcombe et al., 2008). Given the vast increase in molecular understanding of plant biology over the last decade, as well as the successful application of molecular techniques in the field of biomedicine, it has been proposed that the approaches and techniques of molecular biology should be applied to crop improvement strategies in order to increase the abiotic stress tolerance of crops. It is hypothesized that these approaches would generate agronomically useful germplasm with greater speed and precision than classical breeding (Moose and Mumm, 2008; Tester and Langridge, 2010). The first step in any molecular breeding strategy involves defining a suite of candidate genes that possess molecular functions that will enhance the stress tolerance. To date, this has been the rationale of many plant proteomics studies carried out in a wide range of important crop plants. Here, we will critically assess the current state of crop proteomics research and its progress toward the aim of novel gene discovery for abiotic stress tolerance. We will propose a workflow that combines laboratory-based discovery proteomics followed by selected reaction monitoring (SRM) mass spectrometry (MS) of field-grown plants, with the aim of convincing readers that such an approach could contribute more relevant information to advance both gene discovery and gene evaluation for crop improvement programs (**Figure 1**).

## A CRITIQUE OF PROTEOMIC INVESTIGATIONS INTO ABIOTIC STRESS TOLERANCE IN PLANTS

Cellular physiology is underpinned by the composition and function of the proteome, and the power of proteomics techniques lies in their ability to quantitatively analyze large numbers of proteins in parallel, providing rich sets of information with which to explain the molecular mechanisms that underpin cellular function. The plant proteomics literature is characterized by two major threads. The first is the descriptive cataloging of which proteins are expressed in which tissue (Baerenfaller et al., 2011; Lee et al., 2012), localized to which organelle (Heazlewood et al., 2007; Eubel et al., 2008; Taylor et al., 2011), or bound within which protein complex (Remmerie et al., 2011). The second thread is made up of comparative studies that identify differences in protein profiles between tolerant versus sensitive genotypes (Vincent et al., 2007; Jacoby et al., 2010; Pang et al., 2010), or between control plants and plants exposed to environmental stress conditions (Taylor et al., 2005; Kosova et al., 2011). The results from comparative studies are framed as gene discovery exercises, where proteins induced by stress or proteins of higher abundance in the tolerant genotype are positioned as logical candidates for improving stress tolerance in crops, particularly when their molecular functions involve stress related processes such as redox defense or signal transduction. The information gathered by proteomic studies of these kinds has increased the scientific understanding of plant biology, with the results from descriptive proteomic studies being particularly valuable for accurately defining expression profiles across tissue and the subcellular localizations of proteins, while the results of the control versus stress experiments have defined many proteins which are induced in response to stress, significantly increasing our knowledge of how cellular physiology responds to environmental challenge.

However, the value of the above mentioned approaches as gene discovery and ranking strategies for future crop improvement is less clear. Many limitations of the approach and results derived from these experiments must be taken into account before committing to pursue any one protein in a molecular breeding program. First is the common observation that a small subset of the stress responsive proteins are repeatedly identified across a number of different stress experiments, the so called “déjà vu” phenomenon (Petrak et al., 2008). While these proteins undoubtedly play a role in stress response and will be consistently expressed under a wide range of stresses, and their repeated identification might be seen as evidence of their high value, it is likely they will be poor candidate genes for crop improvement, as it is likely that elite agronomic varieties will already increase expression of these proteins in response to stress due to their generalized stress responsiveness. Rather, it is the rarely observed changes in specific experiments that likely hold the key as useful gene traits. A second criticism is the common focus in proteomics papers on the proteins that increased in abundance, over and above those that decreased, and the ranking of fold changes as a proxy for importance. Given the diverse impact of changing the abundance of protein in the control of biochemical pathways, small changes can have large effects and large changes can have small effects, depending very much on the protein in question and its tissue distribution. A third criticism concerns the choice of genotypes and growth conditions used to generate tissue for many of these proteomics studies. To date, proteomics researchers have only sampled a very small portion of the wide diversity that exists between crop varieties, with many experiments repeatedly documenting the stress responses of reference genotypes (e.g., rice cv. Nipponbare, wheat cv. Chinese Spring, barley cv. Golden Promise), which are genetically well characterized, amenable to genetic transformation and have extensive genome sequence resources needed for proteomics identifications, but often show poor agronomic performance. The current focus upon this narrow range of germplasm may limit the range of physiological coping strategies that are being documented. Arguably, this may make the discovery of novel physiological coping strategies rare rather than common events in laboratory settings. More events might be found by analyzing the proteomic responses of diverse varieties adapted to a wider range of environments (Glaszmann et al., 2010). Fourthly, and somewhat similarly to the criticisms leveled at the diversity of genotypes used in studies, there is an obvious difference in timing, severity, and multiplicity of stress responses in plants growth in controlled environments chambers versus crops sown in the field. Most stress proteomic studies analyze the protein expression profiles of plants treated with one single stress under tightly controlled environment growth conditions, with only a smaller number of studies considering two or more stresses, but this contrasts with field-grown plants that will much more routinely experience numerous stresses of varying intensities, which often occur simultaneously (Mittler and Blumwald, 2010). The role of the field environment is particularly important in eliciting genotype × treatment (G × T) interactions, as it has been shown that certain genotypic differences in abiotic stress tolerance only manifest under field conditions (Richards et al., 2010; Tavakkoli et al., 2012). Of course, there are a number of sound reasons which have led proteomics researchers to generate tissue under controlled conditions, such as phenotypic reproducibility, the demand for sufficient quantities of homogeneous tissue, and proximity to laboratory facilities. Furthermore, it is very challenging to conduct reproducible stress treatments in the field to define clear protein targets against a changing background, due to spatial and temporal variations in climate and soils.

We propose that controlled environment experiments that attempt to mimic field conditions are best framed as a starting point to identify proteins of interest. The results of these studies then need to be added together and explored more widely by subsequent profiling experiments conducted under field conditions in the target environment, to determine which, if any, of the favorable molecular mechanisms uncovered in the laboratory hold true in the target environment and what are the relationships between them.

## SELECTED REACTION MONITORING MASS SPECTROMETRY AS A MEANS TO PROFILE THE MOLECULAR MECHANISMS THAT UNDERPIN ABIOTIC STRESS TOLERANCE OF CROPS IN THE FIELD

The ongoing advancement of MS instrumentation and approaches such as sequential window acquisition of all theoretical fragment-ion spectra (SWATH; Gillet et al., 2012) enable current researchers to employ a wider range of methodological approaches. However, these new technologies should not be applied indiscriminately, as it is important to accurately match enhanced technical capabilities and knowledge of their limitations against a relevant biological question in order to generate novel and applicable scientific insights. Here we propose that the emerging plant proteomics application of peptide SRM is well suited to dissecting the molecular mechanisms that underpin phenotypic performance of crop plants in the field. To date this approach has been used to characterize sucrose synthase isoforms and N-metabolism enzymes in *Medicago* (Wienkoop et al., 2008), a basic amino acid carrier involved in arginine metabolism in rice (Taylor et al., 2010) and the plasma membrane transportome in *Arabidopsis* (Monneuse et al., 2011). A number of technical challenges are associated with the establishment of SRM approaches and these have been covered in detail in recent reviews (Picotti and Aebersold, 2012; Thompson et al., 2012). Some of these challenges are particularly pertinent in experiments planned in crop plants. For example it is imperative to determining the uniqueness of a peptide sequence, as valid results are not possible when a peptide used for quantitation is present in more that one protein. In species with well-characterized genomes such as *Arabidopsis* or rice, this can be overcome with computational analysis (Rost et al., 2012). Generally the longer the peptide the more likely it is to be unique. However, in crop plants this remains a challenge, as without complete genome sequences confidence in a peptide’s uniqueness would be limited. Further issues arise when attempting to distinguish between splice variants of a gene or enzyme isoforms. In these cases, it is likely that only very small regions of the proteins differ from one another and thus a limited number of peptides may be available for unique quantitation. Another level of complexity is introduced by the presence of missed cleavages during enzymatic digestion prior to triple-quadrupole (QqQ) MS and the presence of post-translational modification (PTM) sites within peptides selected for SRM. Overall much care must be taken when selecting peptides for quantitation by SRM, particularly in crop plants with incomplete genome sequences and limited knowledge on enzyme isoforms and potential PTMs.

We will highlight the features of SRM that are well matched to the types of biological questions currently being asked by proteomics researchers who seek to define links between protein abundance and abiotic stress tolerance. Firstly the use of a QqQ MS and the implementation of the first quadrupole as a mass filter provides a very high degree of specificity that enables the selective fragmentation and accurate quantitation of peptides with a wide dynamic range, thus enabling low abundance peptides to be detected against complex backgrounds. This feature is advantageous for proteomic analyses of field-grown leaf tissue, where the high abundance of photosynthetic proteins relative to other cellular components means that any analytical technique must be applicable across a large dynamic range. Secondly, SRM methodologies involve the QqQ MS cycling through a pre-defined list of SRM transitions, which ensures that the abundance of each specified peptide will be measured provided it is present in the sample. This contrasts against the “patchy” nature of shotgun proteomics methodologies, where stochastic elements dictate which proteins are documented in any give run, meaning that proteins of biological interest may escape detection across different runs due to random processes. This particular strength of SRM is well matched to the purpose of biomarker validation studies in crops, where the aim is to assess whether the abundance of a specific protein or proteins is correlated to stress tolerance across a given set of genetic material in real field stress scenarios. The data generated by a controlled environment experiment would thus link the abundance of a particular protein to stress tolerance, and the follow-up experiment would involve profiling the abundance of that protein across a large number of field-grown leaf samples to determine the strength and relevance of the correlation. Thirdly, SRM MS coupled to high-performance liquid chromatography (HPLC) is highly suited to high sample throughput of a wider group or groups of proteins in a single analytical run, thus enabling a larger number of data points on different proteins to be gathered per unit of machine time. This is particularly useful for the assessment of protein changes in field-grown crop plants where the variation in the samples may be larger than those collected in the controlled laboratory environment. This also allows the relationships between changes in the abundance of different proteins to be explored in G × T datasets. Usually within the lab, power analysis will reveal that three to five samples are sufficient for the experiment to be informative and this number is amenable to quantitative shotgun proteomic approaches. However, field analysis may require >20 samples for the experiment to be informative and this would led to prohibitively expensive and time-consuming analysis by shotgun proteomic approaches. In these circumstances SRM MS approaches provide an opportunity to quantitate a select group or groups of proteins from a larger number of samples relativity cheaply and quickly. For instance, research suggest that the proteins involved in reactive oxygen species (ROS) detoxification are strongly linked to abiotic stress tolerance (Gill and Tuteja, 2010), but a full understanding of this link is complicated by the fact that the ROS detoxification network in plants involves many proteins spread across numerous cellular compartments (Mittler et al., 2004). Therefore, it can be argued that a SRM MS approach may aid the investigation of which specific ROS detoxification enzymes are causative in abiotic stress tolerance, as this approach would enable not only a large set of ROS detoxification proteins to be profiled in parallel, but it could also be assessed in a wide range of varieties with differing stress tolerance. This is more likely to reveal which specific components of the large ROS detoxification network exhibit consistently higher abundance values in tolerant genotypes than a shotgun proteomic approach.

## DESIRABLE RESOURCES FOR FUTURE SRM MASS SPECTROMETRY STUDIES IN CROP PLANTS

The central difference between SRM MS approaches compared to other proteomics methodologies is the necessity for prior knowledge of proteotypic peptides derived from a protein of interest. Therefore, the most pressing constraint upon SRM approaches in crops is the lack of proteotypic peptides which can be used to quantify proteins of interest in relevant species, meaning that the first step in any SRM investigation involves a long period of library generation, where discovery proteomics and database searching are conducted to define high-quality peptides which are suitable for subsequent SRM experiments. This contrasts with the current state of the SRM field in other species, where for example the SRMAtlas project is collating representative mass spectra for signature peptides derived from tens of thousands of proteins expressed in human, yeast, and mouse (Picotti et al., 2008). This database provides pre-written transition lists for profiling relevant proteins and proteomes (i.e., disease biomarkers in human plasma, central metabolic enzymes in yeast), which can be downloaded and uploaded into the mass spectrometer’s control software without the need for in-house optimization. The rate at which SRM MS approaches are applied to crop plants would likely rapidly increase if a publicly available database that provided high-quality proteotypic peptides and optimized multiplexed SRM transition lists to the community was available.

Despite widespread interest in applying molecular techniques to phenotyping crop plants, difficulties in sequencing the large and complex genomes of many crop species have constrained the power of proteomics applied to crops. DNA sequencing technologies are increasing the speed and decreasing the cost of sequencing, with recent highlights being the generation of reference genomes for barley (Mayer et al., 2012) and tomato (Sato et al., 2012), while rapid progress is being made on the complex wheat genome (Berkman et al., 2012). There is wide variability in abiotic stress tolerance between cultivars (Stone and Nicolas, 1994; James et al., 2008), and different varieties of the same species often exhibit considerable genetic divergence (Gepts, 2006; van de Wouw et al., 2010), so it can be argued that one single “reference” genome per species will not capture the inter-cultivar diversity at the molecular level that manifests in varietal stress tolerance. Studies in wheat cultivars have shown that searching MS results against cultivar-specific sequence databases increases sequence coverage and allows for identification of novel protein isoforms (Altenbach et al., 2010). Therefore, it seems logical that SRM MS studies focusing on varietal differences could derive novel information by profiling the abundance of cultivar-specific protein isoforms, particularly for isoforms of proteins which have been linked to abiotic stress tolerance in different varieties (Sule et al., 2004; Jacoby et al., 2010). A number of initiatives are currently profiling genetic diversity across different varieties of crops (McNally et al., 2009; Lam et al., 2010), and it would be worthwhile for researchers involved in SRM MS studies to develop transitions for the cultivar-specific isoform variants that are documented by these sequencing efforts.

Proteomics research is typically conducted by scientists trained in the disciplines of biochemistry and molecular biology. Their emphasis upon molecular mechanisms is indispensable for understanding the biological meaning of proteomics data. However, due to the specialist knowledge and logistical difficulties inherent in designing and conducting meaningful field experiments, proteomics researchers will likely depend upon collaborations with agriculturally focused researchers in order to access tissue grown in the relevant field environment with appropriate spatial designs and checks. Therefore, building effective collaborations across discipline boundaries is crucial for proteomics researchers who wish to access field-grown material and contribute to crop breeding programs. It can be argued that a mutual appreciation of classical plant physiology is the key bridge between molecular and field researchers, as its emphasis upon dissecting a specific trait at the single plant level is a logical convergence point for ideas and hypotheses stemming from the two different scales (Passioura, 2010). For instance, breeders and agronomists might identify that a given trait (i.e., transpiration efficiency, stem carbohydrate remobilization) leads to a yield improvement under drought conditions, while proteomics researchers can use SRM techniques to investigate the protein abundance profiles of lines which carry this trait, in order to define or validate the proteins that could then serve as candidate genes for breeders. In this way the physiological isolation of a trait which breeders deem to be worthwhile in the target environment can be positioned as a unifying framework which can synthesize the results from molecular studies with agricultural analyses of yield or quality.

## CONCLUSION

As claimed by the introductory sections of many papers, as well as preambles to many grant applications, the strategic endpoint of much plant biology research at the molecular scale is to improve the abiotic stress resistance of crop species. However, much of the data produced by plant proteomics research is yet to be actually evaluated for its use in directing breeding programs. Although the initial identification of candidate proteins linked to stress tolerance will still utilize discovery proteomics workflows applied to plants grown under controlled environment conditions in the lab, the technical capabilities of SRM are a better match for validation studies which aim to quantify the abundance of selected target proteins. The ability of the SRM approach to accurately quantify the abundance of a range of target proteins against complex cellular backgrounds in a large number of field-grown samples is a key to its applicability and value. Therefore, we argue that the judicious development of this SRM approach will further our understanding of the causative links between cellular composition and whole plant stress tolerance, and bring the knowledge and skills of proteomics researchers closer to the stated goal of crop improvement for higher yields in harsh environments.

## Conflict of Interest Statement

The authors declare that the research was conducted in the absence of any commercial or financial relationships that could be construed as a potential conflict of interest.
